# Relationship of postnatal depressive symptoms to infant temperament, maternal expectations, social support and other potential risk factors: findings from a large Australian cross-sectional study

**DOI:** 10.1186/1471-2393-12-148

**Published:** 2012-12-12

**Authors:** John G Eastwood, Bin B Jalaludin, Lynn A Kemp, Hai N Phung, Bryane EW Barnett

**Affiliations:** 1 School of Public Health and Community Medicine, The University of New South Wales, Sydney, NSW, 2052, Australia; 2 School of Women’s and Children’s Health, The University of New South Wales, Sydney, NSW, 2052, Australia; 3 School of Psychiatry, The University of New South Wales, Sydney, NSW, 2052, Australia; 4 Community Paediatrics, South Western Sydney Local Health Network, Hugh Jardine Building, Eastern Campus, Locked Mail Bag 7017, Liverpool, BC NSW, 1871, AUSTRALIA

**Keywords:** Postnatal depression, Temperament, Maternal expectations, Social support, Social isolation, Migrant

## Abstract

**Background:**

From 2000 a routine survey of mothers with newborn infants was commenced in South Western Sydney. The survey included the Edinburgh Postnatal Depression Scale (EPDS). The aim of the study was to determine the prevalence and risk factors for postnatal depressive symptoms in women living in metropolitan Sydney, Australia.

**Methods:**

Mothers (n=15,389) delivering in 2002 and 2003 were assessed at 2–3 weeks after delivery for risk factors for depressive symptoms. The binary outcome variables were EPDS >9 and >12. Logistic regression was used for the multivariate analysis.

**Results:**

The prevalence of EPDS >9 was 16.93 per 100 (95% CI: 16.34 to 17.52) and EPDS >12 was 7.73 per 100 (95% CI: 6.96 to 7.78). The final parsimonious logistic regression models included measures of infant behaviour, financial stress, mother’s expectation of motherhood, emotional support, sole parenthood, social support and mother’s country of birth.

**Conclusions:**

Infant temperament and unmet maternal expectations have a strong association with depressive symptoms with implications for the design of both preventative and treatment strategies. The findings also support the proposition that social exclusion and social isolation are important determinants of maternal depression.

## Background

The impact of maternal depressive illness on subsequent child development is well described [1,2]. There have been extensive studies that have shown that children of mothers who were depressed during infancy are more likely to have behavioural and psychological problems [3-5]. Postnatal maternal depression has also been shown to be associated with impairment of cognitive development and secure attachment [6-8]. Maternal depression is thus a significant public health problem with short and long term impacts on cognitive, emotional, social and behavioural development of infants [8].

The majority of estimates of prevalence in industrialised countries are between 13–20 percent of women [9]. A recent large Australian study found 15.5 percent of postnatal women had depressive symptoms [10]. While the overall prevalence of postpartum depression is similar to other life periods, there is some evidence that there is an increased risk of depression occurring in the early postpartum period [11,12]. Beck undertook a meta-analysis of 84 published studies of postnatal depression and identified 13 significant predictors. They were: prenatal depression, self esteem, childcare stress, prenatal anxiety, life stress, social support, marital relationship, history of previous depression, infant temperament, maternity blues, marital status, socioeconomic status, and unplanned/unwanted pregnancy [13].

Studies of postnatal depression in South Western Sydney have previously been reported [14,15]. From 2000 a routine survey of mothers with newborn infants was commenced. The Ingleburn Baby Information System (IBIS), which includes the Edinburgh Postnatal Depression Scale (EPDS) [16], has been used here to study individual (and family) level factors associated with postnatal depression. The aim is to determine the prevalence and risk factors for postnatal depressive symptoms in women living in metropolitan Sydney, Australia. The study is part of a larger multilevel study that utilises mixed methodology to build a theory describing the mechanisms by which multilevel factors might influence developmental and life course outcomes.

## Methods

### Study design

The study is a population-based cross-sectional study of mothers of infants born in South Western Sydney Area Health Service (SWSAHS) from 2002 to 2003. An exploratory data analysis approach was undertaken for the purpose of informing theory building. The exploratory data analysis included descriptive analysis of data, principal component analysis, and logistic regression. The results of the logistic regression are presented here. Prior knowledge of causal inference was utilised during the analysis to ensure that the variables “conditioned” in the regression analysis were appropriate. The 2002 to 2003 study (n=15,389) is a sub-sample from a larger dataset collected from 1998 to 2006. A 2004 to 2006 subsample was retained for subsequent confirmatory studies.

### Study setting

The setting is in the New South Wales (NSW), Australia, Local Government Areas of Bankstown, Fairfield, Liverpool, Campbelltown, Camden, Wollondilly and Wingecarribee. The area has a diverse multicultural population with 28.4% of the population born overseas compared with 17.8% for the rest of NSW. Twenty percent of infants are born to women from South East, North East or Southern Asia, and approximately one quarter of Sydney’s indigenous population live in South Western Sydney. South Western Sydney is an area of substantial social disadvantage, and has lower education attainment and lower income levels than other parts of NSW.

### Participants and data collection

The study is of 15,389 South Western Sydney mothers who were surveyed, in 2003 and 2004, with the EPDS [16] at the first postpartum home visit. The study utilised the Ingleburn Baby Information System (IBIS) database. This database was initiated in 1995 and is based on the routine survey by Child and Family Health Nurses of all mothers who attend the first well-baby clinic (home visit or clinic-based) after discharge from the post-natal ward. The IBIS database did not collect information on a number of relevant variables, including maternal age and parity, which are routinely collected during pregnancy and childbirth and entered into an obstetric database. Data linkage to obstetric data was possible for the Local Government Areas of Fairfield, Camden, Campbelltown and Wollondilly. This linkage enabled analysis of the relationship of EPDS to maternal age. Population-based collection started in Campbelltown and Wollondilly in 1998, followed by Bankstown in 2000, Fairfield and Wingecarribee in 2001 and Liverpool in 2002. The calendar years of 2002 and 2003 were used for this study as all geographical areas were covered allowing for subsequent individual level, group level and multi-level analysis using the same data set.

The study obtained ethics approval from the Human Research Ethics Committee, South Western Sydney Area Health Service and from the University of NSW Human Research Ethics Committee.

### Outcome variable

EPDS was administered at the time of first well baby visit. Validation studies of the EPDS in English-speaking populations have demonstrated 68% - 86% sensitivity and 78% - 96% [17] and, in an Australian sample, 100% sensitivity and 89% specificity [18]. Positive predictive value for depression has been reported between 70%-90%. Although the drawbacks of using the EPDS in other settings are well recognised, the EPDS has been validated for a number of other languages and ethnic groups. South Western Sydney studies have found that Vietnamese and Arabic translations of EPDS were acceptable to the women and appear to be suitable screening instruments for postnatal distress and depression in these populations [14,15]. The EPDS has not been validated for Aboriginal and Torres Strait Islander Peoples, although it is widely used in these populations in urban settings.

This study reports on two outcome variables, namely EPDS >9 and EPDS >12. Both EPDS >9 and EPDS >12 are supported by previous studies as screening cut-off points in English-speaking populations. Buist and colleagues [19] note that using EPDS >12 is a sound option for reducing false positive diagnosis of depressive illness. Using EPDS >9 as a community screening cut-off point is recommended by the original authors of the scale as many women who experience considerable dysfunction but might not meet diagnostic criteria for formal illness, might otherwise be missed. These women also merit recognition of their distress and provision of psychological or social assistance [19,20]. In this study the EPDS was administered to non-English speaking mothers through interpreters.

### Exposure variables

The IBIS survey contains 45 items which are both clinical (e.g. weight) and parental self-report in nature. Forty variables were selected for exploratory analysis based on prior knowledge, the findings of published research and the findings of the qualitative arm of the study. The exposure variables selected for study were: mother’s country of birth (Australia or other), Aboriginal or Torres Strait Islander culture, age of mother, sex of baby, marital status, household size, blended family, number of children under five years of age, accommodation (privately owned or not), employment of mother, employment of father, financial situation (10-point scale), car access, phone access, mother’s rating of her health (five-point scale), mother’s rating of her child’s health (five point scale), breastfeeding (which included both exclusive and partial breastfeeding), smoking, mother’s expectations (“Is being a mother what you expected” – five-point scale), planned pregnancy, previous miscarriage, previous child death, previous stillbirth, previous child disability, previous termination of pregnancy, previous sudden infant death, suburb duration, regret about leaving the suburb (“If for some reason you had to leave this suburb would you be sorry to go?”), support network (“If you had any worries about your child, how many people do you feel you could turn to for help and support, not including health professionals?”), practical support (“Do you receive adequate practical support since the birth of the baby?), emotional support (“Have you been able to talk to someone about how you are feeling since the birth of the baby?”), mother’s response to her child (“Does the mother respond to the child’s interactions of discomfort?”), mother comforts her child (“Does the mother show the ability to comfort the child?”), mother enjoys contact with the baby (“Does the mother enjoy close physical contact with the child?”), and “Since the birth of your baby how much time did your baby seem: - to have trouble sleeping (five-point scale), to be a demanding baby (five-point scale), to be content (five-point scale), to be a difficult feeder (five-point scale), or to be difficult to comfort (five-point scale)”.

### Causal models

Care should be taken in regression studies to avoid controlling for a variable that is a descent (effect) of the outcome under study. Adjustment for factors that might be on the causal pathway between the exposure and the outcome is also often unwarranted [21]. Prior to undertaking regression analysis it is therefore important to state the hypothetical causal networks being proposed. Pearl [22] proposed a graphical method of selecting a set of factors for adjustment. The so called “back-door” criterion states that a set S is appropriate for adjustment if two conditions hold: 1) no element of S is a descent of X, and 2) the elements of S “block” all “back-door” paths from X to Y, namely all paths that end with an arrow pointing to X. In an exploratory study such as the one described here it remains necessary to limit the possibility of spurious findings. Diagrams known as directed acyclic graphs (DAGs) were used to make explicit why variables were entered, or not entered, into the logistic regression.

### Statistical analysis

The exploratory data analysis included uni-variate analysis of the exposure variables against the outcome variables. For the dichotomous outcomes the relationship with nominal and ordinal variables was assessed using Pearson chi-square tests and uni-variate logistic regression. Estimates of odds ratios and confidence intervals were made. For the ordinal variables scatter plots were also undertaken. Exploratory factor analysis was undertaken (not reported here) using Categorical Principal Components Analysis (CatPCA) to identify underlying common factors and to inform the causal modeling.

Logistic regression was used for the multivariate analysis and is reported here. Both the EPDS >9 and EPDS >12 dichotomous outcomes were modeled. Candidate variables were selected for the multivariate model if the uni-variate test had a *p*-value <0.25 or was known to be biologically or theoretically important. As described by Hosmer and Lemeshow (2000) the first model contained all the selected variables. Following the initial fit of the multivariate logistic models the importance of each variable was verified. This was undertaken by examination of the Wald statistic for each variable and comparison of each estimated coefficient with the coefficient from the uni-variate model containing only that variable. The process of deleting, refitting and verifying continued manually until we were satisfied that the important variables were included in the model.

Continuous variables were checked for linearity in the logit. Interaction terms were created and tested in the model for improved model fit. Multicolinearity was assessed through analysis of the correlation coefficients in the Logistic Regression Correlation Matrix and inspection of the standard error for each variable. The model fit was assessed using the Hosmer-Lemeshow goodness of fit [23] and plotting of the predicted probabilities using the receiver operating characteristics (ROC). Diagnostic assessment used plots of 1) Deviance of studentized residuals versus estimated Logistic Probabilities, and 2) Cooks distances versus estimated Logistic Probabilities. All analyses were undertaken using SPSS 18.0.

## Results

Of the 15,389 infants and mothers in the sample population, 45.3 percent of the mothers were born in a country other than Australia and 1.9 percent had an Aboriginal background. The majority of mothers (75.5%) were married, 15.5 percent lived with a partner and 9 percent were single. Of the women interviewed, 13.9 percent had blended families. Of note, 16.2 percent of women lived in large households with more than five members.

Six percent lived in “disadvantaged” accommodation, which included public housing, caravans or refuges. Almost eight percent of women described their financial status as difficult (0–3 on a scale of 10).

Of the mothers interviewed, 33.4 percent reported that their pregnancy was unplanned and the majority of mothers (91%) had two or fewer children under five years of age. Approximately 10 percent had no access to a car and two percent did not have access to a phone.

Maternal age was available (by linkage to obstetric data) for women living in Fairfield, Campbelltown, Camden and Wollondilly LGA. Of 6,900 women, 92 percent were aged between 20–39 years of age, six percent were less than 20 years of age and two percent forty years of age or greater.

The mean EPDS Score was 5.48 (95% CI: 5.41 to 5.55). The mean postpartum period was 3.77 weeks (95% CI: 3.62 to 3.92). The prevalence of EPDS >9 was 16.93 per 100 (95% CI: 16.34 to 17.52) and EPDS >12 was 7.73 per 100 (95% CI: 6.96 to 7.78).

Chi Square analysis identified the following as not being significantly associated with either EPDS >9 or >12: sex of the baby, being Aboriginal or a Torres Strait Islander; smoking, education of mother, age of mother, previous miscarriage, previous child death, previous stillbirth, previous pregnancy termination, previous sudden infant death or previous child disability. Those variables were not analysed further (note that the EPDS has not been validated for use with Aboriginal or Torres Strait Islander peoples). Chi square analysis and uni-variate logistic regression of all other study variables were significantly associated with the EPDS outcome measures. Uni-variate (unadjusted) and Multi-variate (adjusted) logistic regression results are presented in Tables 1 and 2 for EPDS >9 and >12 respectively.

**Table 1:** Uni-variate (unadjusted) and Multi-variate (adjusted) logistic regression - EDS >9

	**Unadjusted**			**95% CI of OR**		**Adjusted**			**95% CI of OR**	
**Variable**	**B**	**SE**	**OR**	**Low**	**Upper**	**B**	**SE**	**OR**	**Low**	**Upper**
**Demographic**										
Country of Birth (D)	0.504	0.047	1.656	1.511	1.814	0.235	0.066	1.264	1.112	1.438
Sole Parent (D)	0.399	0.072	1.490	1.293	1.717	0.018	0.144	1.018	0.768	1.349
Household Size (C)										
Less than six			1.00					1.00		
Six to ten	−0.055	0.063	0.947	0.837	1.071	−0.072	0.085	0.931	0.789	1.099
Greater than ten	0.671	0.260	1.957	1.174	3.260	0.440	0.370	1.553	0.752	3.207
Blended Family (D)	0.199	0.065	1.221	1.074	1.387	0.066	0.090	1.068	0.895	1.273
**Socioeconomic**										
Accommodation (D)	0.266	0.089	1.304	1.096	1.552	−0.135	0.141	0.874	0.662	1.153
Unemployed Father (D)	0.200	0.089	1.221	1.026	1.454	−0.202	0.122	0.817	0.643	1.038
Financial Situation (S)	0.215	0.015	1.239	1.204	1.276	0.094	0.020	1.098	1.057	1.142
Car Access (C)										
Always			1.00					1.00		
Occasionally	0.242	0.061	1.273	1.130	1.434	−0.297	0.087	0.743	0.626	0.882
Never	0.506	0.064	1.659	1.463	1.882	0.116	0.094	1.123	0.933	1.351
**Health**										
Mothers Health (S)	0.594	0.031	1.812	1.706	1.923	0.371	0.044	1.449	1.328	1.581
Child's Health (S)	0.383	0.031	1.467	1.382	1.558	−0.036	0.045	0.964	0.883	1.053
Mothers Expectations(S)	0.851	0.030	2.342	2.207	2.484	0.629	0.038	1.875	1.740	2.021
**Pregnancies**										
Planned Pregnancy (D)	0.292	0.047	1.339	1.220	1.469	0.148	0.066	1.159	1.019	1.319
**Social Network**										
Suburb Duration (D)	0.224	0.048	1.251	1.139	1.374	0.079	0.065	1.083	0.953	1.229
Regret Leaving Suburb (D)	0.274	0.053	1.316	1.186	1.459	0.092	0.071	1.096	0.953	1.260
Support Network (S)	0.405	0.021	1.499	1.439	1.562	0.137	0.032	1.147	1.078	1.221
Practical Support (D)	0.756	0.056	2.129	1.909	2.375	0.232	0.084	1.262	1.070	1.487
Emotional Support (D)	1.064	0.063	2.898	2.560	3.282	0.472	0.093	1.603	1.336	1.924
**Baby Characteristics**										
Baby Trouble Sleeping (S)	0.434	0.027	1.544	1.464	1.628	0.149	0.047	1.161	1.058	1.274
Baby Demanding (S)	0.378	0.024	1.460	1.392	1.531	0.090	0.042	1.094	1.008	1.188
Baby Content (S)	0.471	0.035	1.602	1.496	1.716	0.177	0.053	1.193	1.075	1.325
Baby Difficult Feeder (S)	0.286	0.030	1.332	1.256	1.412	0.034	0.043	1.035	0.950	1.127
Baby Difficult to Comfort (S)	0.405	0.03	1.499	1.414	1.589	0.031	0.051	1.032	0.935	1.139
Health of Child (S)	0.383	0.031	1.467	1.382	1.558	−0.036	0.045	0.964	0.883	1.053

(D) – dichotomous, (C) – categorical, (S) - scale.

**Table 2:** Uni-variate (unadjusted) and Multi-variate (adjusted) logistic regression - EDS >12

	**Unadjusted**			**95% CI for OR**		**Adjusted**			**95% CI for OR**	
**Variables**	**B**	**SE**	**OR**	**Lower**	**Upper**	**B**	**SE**	**OR**	**Low**	**Upper**
**Demographics**										
Country of Birth (D)	0.544	0.064	1.722	1.519	1.953	0.096	0.097	1.101	0.909	1.332
Sole Parent (D)	0.711	0.086	2.036	1.722	2.408	0.240	0.185	1.271	0.884	1.828
Household Size (C)										
Less than Six				1.00				1.00		
Six to ten	0.040	0.083	1.041	0.885	1.225	0.048	0.121	1.049	0.828	1.329
Greater than ten	0.968	0.290	2.632	1.490	4.651	0.919	0.441	2.506	1.055	5.951
Blended Family (D)	0.307	0.084	1.359	1.154	1.600	0.099	0.127	1.104	0.860	1.416
**Socioeconomic**										
Accommodation (D)	0.366	0.109	1.441	1.164	1.784	−0.234	0.202	0.791	0.533	1.175
Unemployed Father (D)	0.271	0.116	1.311	1.044	1.647	−0.195	0.171	0.823	0.589	1.150
Financial Situation (S)	0.245	0.020	1.277	1.229	1.328	0.129	0.029	1.138	1.076	1.204
Car Access (C)										
Always				1.00				1.00		
Occasionally	0.357	0.081	1.428	1.220	1.673	−0.103	0.122	0.902	0.711	1.146
Never	0.636	0.082	1.890	1.611	2.217	0.194	0.130	1.214	0.941	1.565
**Health**										
Mother’s Health (S)	0.704	0.042	2.021	1.863	2.193	0.492	0.064	1.635	1.443	1.853
Child’s Health (S)	0.385	0.042	1.470	1.355	1.595	−0.121	0.065	0.886	0.780	1.006
Mothers Expectations (S)	1.019	0.042	2.771	2.550	3.012	0.774	0.056	2.169	1.944	2.420
**Pregnancies**										
Planned Pregnancy (D)	0.469	0.063	1.599	1.413	1.809	0.165	0.095	1.179	0.978	1.421
**Social Network**										
Suburb Duration (D)	0.362	0.067	1.436	1.259	1.638	0.102	0.094	1.107	0.921	1.330
Regret Leaving Suburb (D)	0.492	0.069	1.635	1.429	1.872	0.202	0.101	1.224	1.005	1.491
Support Network (S)	0.401	0.026	1.493	1.419	1.571	0.158	0.043	1.171	1.077	1.275
Practical Support (D)	0.708	0.071	2.031	1.768	2.333	0.250	0.115	1.284	1.026	1.607
Emotional Support (D)	1.045	0.076	2.844	2.449	3.303	0.569	0.122	1.766	1.391	2.243
**Baby Characteristics**										
Baby Trouble Sleeping (S)	0.406	0.035	1.501	1.402	1.609	0.180	0.067	1.197	1.049	1.365
Baby Demanding (S)	0.392	0.031	1.479	1.392	1.572	0.164	0.058	1.179	1.051	1.321
Baby Content (S)	0.390	0.045	1.477	1.352	1.614	0.163	0.077	1.177	1.012	1.369
Baby Difficult Feeder (S)	0.208	0.039	1.231	1.140	1.330	−0.019	0.061	0.981	0.870	1.106
Baby Difficult to Comfort (S)	0.458	0.039	1.581	1.463	1.707	−0.029	0.070	0.972	0.847	1.114
Health of Child (S)	0.385	0.042	1.470	1.355	1.595	−0.121	0.065	0.886	0.780	1.006

(D) – dichotomous, (C) – categorical, (S) - scale.

### Mother’s expectation

Mother’s expectation of motherhood was highly associated with EPDS >9 (OR 2.34; 95% CI: 2.2-2.5) and EPDS >12 (OR 2.77; 95% CI: 2.55-3.00). When adjusted for all other study factors the association remained strong - EPDS >9 (OR 1.88; 95% CI: 1.74 – 2.02) and EPDS >12 (OR 2.17; 95% CI: 1.94 – 2.42). Maternal expectation remained significant in the final main effects parsimonious models presented in Table 3 and 4 - EPDS >9 (OR 1.90; 95% CI: 1.78 – 2.04) and EPDS >12 (OR 2.13; 95% CI: 1.93 – 2.35).

**Table 3:** Final Parsimonious Model EDS >9

	**B**	**S.E.**	**Wald**	**df**	**Sig.**	**OR**	**95% C.I. for OR**	
							**Lower**	**Upper**
Maternal Expectation(S)	0.642	0.035	338.182	1	0.000	1.901	1.775	2.036
Baby Difficult Sleep(S)	0.138	0.042	11.032	1	0.001	1.148	1.058	1.246
Baby Demanding(S)	0.092	0.038	5.975	1	0.015	1.096	1.018	1.180
Baby Not Content(S)	0.171	0.048	12.534	1	0.000	1.186	1.079	1.304
Practical Support(D)	0.205	0.075	7.481	1	0.006	1.228	1.060	1.422
Emotional Support(D)	0.446	0.083	29.109	1	0.000	1.562	1.328	1.837
Social Support(S)	0.149	0.028	28.040	1	0.000	1.161	1.099	1.227
Financial Situation(S)	0.086	0.017	25.094	1	0.000	1.090	1.054	1.127
Country of Birth(D)	0.225	0.058	15.070	1	0.000	1.252	1.118	1.402
Mothers Health(S)	0.365	0.037	98.047	1	0.000	1.440	1.340	1.548
Constant	−6.216	0.178	1213.109	1	0.000	0.002		

(D) – dichotomous, (S) - scale.

**Table 4:** Final Parsimonious Model EDS >12

	**B**	**S.E.**	**Wald**	**df**	**Sig.**	**OR**	**95% C.I. for OR**	
							**Lower**	**Upper**
Mothers Expectation(S)	0.757	0.050	229.866	1	0.000	2.131	1.933	2.350
Baby difficult sleep(S)	0.148	0.056	6.900	1	0.009	1.160	1.038	1.295
Baby Demanding (S)	0.155	0.050	9.697	1	0.002	1.167	1.059	1.287
Sole Parent (D)	0.444	0.114	15.282	1	0.000	1.559	1.248	1.947
Emotional Support(D)	0.575	0.100	33.012	1	0.000	1.777	1.461	2.162
Social Support(S)	0.203	0.037	30.695	1	0.000	1.226	1.141	1.317
Financial Situation(S)	0.132	0.025	27.694	1	0.000	1.141	1.086	1.199
Maternal Health(S)	0.460	0.053	74.259	1	0.000	1.583	1.426	1.758
Country of Birth(D)	0.091	0.084	1.174	1	0.279	1.095	0.929	1.291
Constant	−8.051	0.248	1054.068	1	0.000	0.000		

Legend: (D) – dichotomous, (S) - scale.

### Maternal support

Measures of support for mothers were all significant in the univariate analysis including: duration living in the suburb, having regret if leaving the suburb, size of social network, and access to emotional and practical support. Suburb duration and regret leaving did not remain significant. Of the remaining three support variables, emotional support was strongly associated - EPDS >9 (OR 1.60; 95% CI: 1.34 – 1.92) and EPDS >12 (OR 1.76; 95% CI: 1.39 – 2.24). Emotional support, practical support and social support all remained significant in the final main effects parsimonious model for EPDS >9, while social support and emotional support remained significant for EPDS >12. Being a sole parent was not significant for EPDS >9 when adjusted but remained so for EPDS >12 in the final main effects model (OR 1.56; CI: 1.25 – 1.95).

### Country of birth

Mothers not born in Australia were more likely to have depressive symptoms with EPDS >9 (OR 1.66; 95% CI: 1.51 – 1.81) and EPDS >12 (OR 1.72; 95% CI: 1.52 – 1.95). When adjusted for all other study factors the association only remained for EPDS >9 (OR 1.26; 95% CI: 1.11 – 1.43). The association was stronger in the final main effects model for EPDS >9 (OR 1.9; 95% CI 1.78 – 2.04). While not statistically significant this variable improved the final main effects model fit for EPDS >12 and was therefore retained in the model.

### Infant temperament

All five variables related to infant temperament were significant in the uni-variate analysis including: baby having trouble sleeping, baby being demanding, baby not being content, baby being a difficult feeder and baby being difficult to comfort. Baby being a difficult feeder or difficult to comfort were not significant when adjusting for the other variables. Baby having trouble sleeping remained associated in the final main effects parsimonious model for both outcomes - EPDS >9 (OR 1.15; 95% CI: 1.06 – 1.25) and EPDS >12 (OR 1.16; 95% CI: 1.04 – 1.23). Baby being demanding also remained associated in the final model - EPDS >9 (OR 1.01; 95% CI 1.02 – 1.18) and EPDS >12 (OR 1.17; 95% CI: 1.06 – 1.29). Baby not being content only remained significant in the final model for EPDS >9 (OR 1.19; 95% CI: 1;08 – 1.30).

### Socioeconomic circumstances

Of the several measures of socioeconomic circumstances only self-reported financial stress remained significant when controlling for the other factors. The association was not strong in the final models - EPDS >9 (OR 1.09; 95% CI 1.05 – 1.13) and EPDS >12 (OR 1.14; 95% CI: 1.09 – 1.19).

### Maternal self-reported health

Maternal health (self-report) was associated with EPDS >9 (OR 1.81; 95% CI: 1.71 – 1.92) and EPDS >12 (OR 2.02; 95% CI: 1.86 – 2.19). When adjusted for all other study factors the association remained strong - EPDS >9 (OR 1.45; 95% CI: 1.33 – 1.58) and EPDS >12 (OR 1.64; 95% CI: 1.44 – 1.85). Maternal health remained significant in the final main effects parsimonious models presented in Tables 3 and 4 - EPDS >9 (OR 1.44; 95% CI: 1.34 – 1.55) and EPDS >12 (OR 1.58; 95% CI: 1.43 – 1.76).

### Final model EPDS >9

For the Final Parsimonious Model EPDS >9, (Table 3), the Hosmer and Lemeshow Test was not significant (*Χ*^2^ 3.682, df 8, p =0.885) indicating that the data fit the model well. The model was able to correctly classify 98.1% of EPDS >9 for an overall success rate of 83.2%. The area under the ROC curve was 74 (95% CI: 72.7 - 75.2) indicating an adequate fit.

### Final model EPDS > 12

For the Final Parsimonious Model EPDS >12, (Table 4), the Hosmer and Lemeshow Test was a not significant Chi-square (*X*^2^ 2.921 df 8, p =0.939) indicating that the data fit the model well. The model was able to correctly classify 99.6% of EPDS >12 for an overall success rate of 92.5%. The area under the ROC curve was 78.5 (95% CI: 76.8 – 80.1) indicating an adequate fit. Mother’s Country of Birth was not significant but its inclusion in the model improved the fit to a Hosmer and Lemeshow Test of p=0.939.

## Discussion

The cross-sectional logistic regression reported here was undertaken as part of a multilevel mixed method theory building study in a large Sydney multicultural population. Our findings were consistent with previous individual level risk factor studies with: financial difficulties, lack of emotional support, maternal expectations of motherhood, maternal self-reported health, infant temperament, and lack of social support, sole parenthood, and maternal migration being predictors of self-reported depressive symptoms. The prevalence of EPDS >9 (16.9%) and >12 (7.7%) at a mean postpartum age of 3.7 weeks was similar to other New South Wales studies [24]. In addition, the predictors identified here were consistent with thematic concepts emerging from the qualitative arm of our study including: loss of expectations and dreams, marginalisation and “being alone”, lack of support and nurturing, and loss of power and control.

### Social support

Our study confirmed a strong association between lack of support and maternal depressive symptoms. “Social support” has been broadly defined as “resources provided by others” and as “the emotional, instrumental, or financial aid” that is obtained from one’s social relationships [25]. Sources of support can be a spouse, relatives, friends, or associates and there are different types of social support, e.g., *informational* support (where advice and guidance is given), *instrumental* support (practical help in terms of material aid or assistance with tasks), and *emotional* support (being a confidant, expressions of caring and esteem). In our study the following measures, where support may have played a role, were significant in the unadjusted analysis: mother not born in Australia, sole parenthood, duration living in the suburb, having regret leaving the suburb, size of social network and access to emotional and practical support. Sole parenthood, mother not born in Australia, lack of a social support network and lack of emotional support remained significant in the final parsimonious models. The measure of emotional support was strongly associated in the final models. The strength of the association with emotional support is surprising given that 91% of mothers reported having a partner. Relationship difficulties have previously been reported to be strongly associated with postnatal depression and the measure of emotional support used here may have been measuring relationship difficulties.

Social support has consistently been found to be associated with perinatal depressive symptoms. In her most recent systematic review of predictors of postnatal depression Beck [13] identified 27 studies that examined social support. The relationship between social support and postpartum depression had a moderate effect size. Studies have also consistently shown a negative correlation between postpartum depression and lack of emotional and instrumental support [13].

### Not born in Australia

Our finding of increased depression among mothers not born in Australia is consistent with the protective effective of social support networks. Immigrants can face increased stressors related to discrimination or the stress of adjusting to a new culture. Social support might be particularly relevant in that context. Perinatal depression has been found to be more common among recent migrants to Australia [26]. Small and colleagues found that the rates were high among Turkish women but relatively low among Vietnamese and Filipino women [27]. Of direct relevance to this study are the findings of Stuchbery and colleagues [28] who undertook a study of Vietnamese, Arabic-speaking and Anglo-Celtic mothers in South West Sydney specifically to examine which deficits in their social support network were associated with postnatal depression. For Anglo-Celtic women, low postnatal mood was associated with a perceived need for more emotional support from partners and mothers, and for Vietnamese mother’s low mood was associated with poor quality relationship with the partner and a perceived need for more practical support from him. For Arabic-speaking women, low mood was associated with a perceived need for more emotional support from partners.

### Maternal expectations

Our survey included the question “is being a mother what you expected?” which was coded on a five point Likert scale. The strong association in this analysis of maternal expectations with depressive symptoms is consistent with previous studies where this has been examined. Beck’s metasynthesis of 18 qualitative studies of postpartum depression which identified “incongruity between expectations and the reality of motherhood” as one of four perspectives of postnatal depression [29]. The finding is also consistent with the findings of our qualitative study. We have not reported here on the relationship between maternal expectations and other covariates such as infant temperament. It is plausible that maternal expectations affect the mothers’ perception of her infants’ temperament or it may influence her response to her infant’s needs.

### Infant temperament

We found strong associations among various measures of infant behaviour and maternal depressive symptoms. The baby having difficulty sleeping, and being demanding, remained significant in the final models for both EPDS >9 and >12. These findings may be related to direct and interactive effects of: 1) the impact of maternal stress and mood on the infant, 2) the impact of the infant’s behaviour on the mother, and 3) mothers perception of her infant’s behaviour. While antenatal stress and depression have been shown to have an impact on infant behaviour and attachment [30], our concurrent qualitative research suggested that, at least as perceived by the mother, poor infant sleeping was a cause of maternal stress and depression. During the causal preparation of DAGs we therefore elected to include the infant temperament variables in the logistic regression.

Beck, in her 2001 systematic review found that infant temperament was moderately related to postpartum depression. The mean *r* effect size ranged from .33 to .34 [13]. This finding was consistent with her earlier systematic review [31]. Murray et al. [32] followed a group of women from the third trimester of pregnancy until the infant’s second birthday. When the infants were 10 days old they tested for infant irritability and maternal depressive symptoms. Mothers with depressive symptoms were dropped from the study and the remaining mothers assessed again at 6, 8 and 18 weeks. Neonatal irritability was the best predictor of the mothers developing depression in a group of women who were at high risk of developing postnatal depression. Infant irritability was not a risk factor among low risk mothers [32]. In our study the direction of the association remains uncertain. It is certainly plausible that in the absence of support mothers will be more sleep deprived than with good support. Infants are so finely tuned to maternal mood that irritability will develop if they are not generously soothed.

### Financial stress

Our study found associations of depressive symptoms with several measures of poverty and social exclusion including: financial difficulties, accommodation, father’s unemployment, sole parenthood and access to a car. Self-reported financial difficulties remained significant in both the final models and sole parenthood in the final EPDS >12 model. This association of financial difficulties with postnatal depressive symptoms has been found in previous studies [33,34]. Beck did not find SES to be significant in her first meta-analysis [31] but added both SES and unplanned pregnancy in her update [13]. The relationship between SES and postpartum depression, in the 2001 published meta-analysis, was in the range of a small effect size (0.19 ~ 0.22). There is increasing evidence for contextual effects of area-level economic deprivation on mental health [35,36]. A qualitative study of pathways from neighbourhoods to mental well-being found that neighbourhood affordability, negative community factors including crime and vandalism, and social makeup including unemployment and poverty, were felt to be associated with poor mental well-being [37].

### Maternal self-reported health

In our study mothers were asked “in general how would you rate your own health?” This was a five-point Likert scale. Maternal self-rated health was strongly associated with depressive symptoms and remained significant in both the main effects models. Self-rated health is interpreted as a global measure of health and well-being [38]. A study of new mothers in Sweden found factors associated with poor self-rated health included tiredness, musculoskeletal problems, abdominal pain, emotional problems, depression, negative experiences of breast feeding, infant sleeping problems, prematurity and poor social support [39]. Postnatal depression has previously been found to be associated with poor self-rated health [40]. It is also possible that poor self-rated health is related to the feeling of “loss of control” and to birth-related trauma and stress.

### Methodological issues

The size (15,389) of this cross-sectional study of the EPDS administered to postnatal women is unique. There have been few previous reports of postnatal depression studies on population samples of this order. Ferguson et al. [12] interviewed 9,316. Most other large studies have been of samples less than 3,000 women [41-46]. The prevalence of EPDS score >9 (16.9%) and >12 (7.7%) is similar to that found in other studies [24,47]

The cross-sectional design of this study has limitations in relation to drawing inferences from the regularities observed. The direction of the relation cannot be established in cross-sectional quantitative studies. Criteria for inferring a causal relationship in such situations include those proposed by Hill [48]. Cross-sectional quantitative studies are also unable to identify the course of disease or symptoms over time. It could be that the disease was caused by some prior event not captured in the cross-sectional design. Maternal depression may have existed prior to the pregnancy and be unrelated to the regularities observed. The study used secondary data sources and the independent variables available for study were limited to those included in the IBIS self-report survey. Consequently we were unable to report on important variables that might have been included if the survey had been specifically designed for the study of perinatal and postpartum depression. Significantly there was limited information on personality traits, psycho-pathological factors, life events and lifestyle behaviours.

Selection bias may have occurred from refusal and non-response in the study population. Importantly not all households with births in the study period were surveyed. The households not questioned with the IBIS questionnaire include mothers who moved to “out of area” locations or mothers who declined the nurse first-visit offered. The population who refused an early childhood nurse visit may represent a particular socio-demographic sample.

Observational (information) bias may have been present in the survey data. This could have arisen from recall bias, or interviewer / responder bias. A particular problematic feature of self-reporting surveys is the mental state of the subject. Depressed women are more likely to have a negative view of their circumstances. This must be taken into account when considering the association found in this study of high EPDS with subjective variables such as “rating of own health”, “reluctance to leave the suburb”, and “difficult financial situation”.

The EPDS is administered in English or via an interpreter where the mother is non-English speaking (NESP). We were not able to report in this study on the percentage of NESP mothers in the full sample but the percentage of NESP mothers in the Local Government Areas of Fairfield, Campbelltown, Camden and Wollondilly (linked data) was 11.8 percent at the time of the 2001 Census. This may be an important source of information bias in this study. Specific non-English EPDS are not currently used postpartum in SWS but could be considered for future use.

The mean time of administration of the EPDS was 3.77 weeks postpartum. This is earlier than most other studies which administer assessments in the first 6 – 12 weeks after delivery. The results could potentially be influenced by “Baby/Maternity Blues” (3 – 10 days postpartum) in some respondents with an overestimate of the prevalence of difficulties.

### Implications

The study reported here confirms the importance of poverty (“difficult financial circumstances”) and isolation (sole parenthood and lack of emotional and social support) as independent risk factors for maternal depressive symptoms. Taken together these findings support the proposition that social exclusion and social isolation are important determinants of maternal dysphoria with implications for both preventative and treatment strategies. The higher rates of depressive symptomatology among mothers not born in Australia may also be related to social isolation and social exclusion as part of the acculturation process.

The finding of a strong association of depressive symptoms with unmet maternal expectations confirms findings of earlier studies [49]. The strong association of infant behaviour at a mean age of 3 weeks with depressive symptoms supports the proposition that infant irritability is a cause of maternal stress and depression. The possibility of reverse or two-way association cannot, however, be excluded. Interventions that seek to address antenatal maternal expectations of infant behaviour, infant soothing techniques, and relationship adjustment and provide support in the early postnatal period may prove to be effective interventions.

The long term consequence of perinatal depression indicates that preventive interventions for perinatal depression are necessary and important. Both intensive professional postpartum support [50] and home visiting [51] have been confirmed as effective interventions for postnatal depression. Social support networks were protective in the study reported here suggesting that antenatal interventions that promote friendship groups may be beneficial. The effectiveness of antenatal groups in preventing postnatal depression has not yet been confirmed [52] but proactive telephone based peer support has been found to be protective [53]. Thus the findings from this study and recent intervention studies indicate that there is merit in maternal and child health services continuing to develop and evaluate interventions that identify and provide early support for mothers who have, or are “at risk” of, developing depression.

## **Conclusions**

Infant temperament and unmet maternal expectations have a strong association with depressive symptoms with implications for the design of both preventative and treatment strategies. The findings reported here also support the proposition that social exclusion and social isolation are important determinants of maternal depression. The detrimental or protective impact of group-level factors on maternal depression have not been examined here. We plan to use the information from this study to inform future multilevel and spatial regression studies. Cross sectional studies and regression analysis, as used in this study, cannot adequately examine causal paths or the effects of mediation and moderation. Path analysis and structural modelling using a longitudinal data set will be required for this purpose.

## Abbreviation

EPDS: Edinburgh Postnatal Depression Scale; IBIS: Ingleburn Baby Information System; NSW: New South Wales; DAGs: Directed acyclic graphs; SWSAHS: South Western Sydney Area Health Service.

## Competing interests

The authors declare that they have no competing interests.

## Author’ contributions

JGE made the study designs, conceptualised the report, did the data analysis, data interpretation, and wrote the report. BBJ, LAW, HNP, BEWB made critical and technical contribution to the study design, analysis and report writing. All authors read and approved the manuscript.

## Pre-publication history

The pre-publication history for this paper can be accessed here:

http://www.biomedcentral.com/1471-2393/12/148/prepub
